# Role of Lipoprotein Ratios and Remnant Cholesterol in Patients with Myocardial Infarction with Non-Obstructive Coronary Arteries (MINOCA)

**DOI:** 10.3390/jcdd11050146

**Published:** 2024-05-08

**Authors:** Vincenzo Sucato, Luca Di Fazio, Cristina Madaudo, Giuseppe Vadalà, Alessandro D’Agostino, Salvatore Evola, Giuseppina Novo, Egle Corrado, Alfredo Ruggero Galassi

**Affiliations:** Department of Health Promotion, Mother and Child Care, Internal Medicine and Medical Specialties (PROMISE), University Hospital “Paolo Giaccone”, University of Palermo, 90133 Palermo, Italy

**Keywords:** MINOCA, remnant cholesterol, lipoprotein ratio, dyslipidemia

## Abstract

Background: Myocardial infarction with non-obstructive coronary arteries (MINOCA) is a clinical situation characterized by evidence of acute myocardial infarction (AMI)—according to the Fourth Universal Definition of Myocardial Infarction—with normal or near-normal coronary arteries on angiographic study (stenosis < 50%). This condition is extremely variable in etiology, pathogenic mechanisms, clinical manifestations, prognosis and consequently therapeutic approach. Objective: The objective of the study was the evaluation of remnant cholesterol (RC), monocyte/high-density lipoprotein cholesterol ratio (MHR), platelet/lymphocyte ratio (PLR) and various lipoprotein ratios in patients with MINOCA in order to establish their validity as predictors of this event. Materials and Methods: We included 114 patients hospitalized in the Intensive Coronary Care Unit (ICCU) and Hospital Wards of our Hospital Center from 2015 to 2019 who received a diagnosis of MINOCA compared to a control group of 110 patients without previous cardiovascular events. RC was calculated with the following formula: RC = total cholesterol (TC) − HDL-C − LDL-C. MHR was calculated by dividing the monocyte count in peripheral blood by high-density lipoprotein cholesterol (HDL-C) levels; PLR was obtained by dividing platelet count by lymphocyte count. We also calculated various lipoprotein ratios, like total cholesterol/high-density lipoprotein cholesterol (TC/HDL-C), low-density lipoprotein cholesterol/high-density lipoprotein cholesterol (LDL-C/HDL-C), triglycerides/high-density lipoprotein cholesterol (TG/HDL-C), and non-high-density lipoprotein cholesterol/high-density lipoprotein cholesterol (non-HDL-C/HDL-C) ratios. Results: The MINOCA group had higher mean levels of RC (21.3 ± 10.6 vs. 13.2 ± 7.7 mg/dL), MHR (23 ± 0.009 vs. 18.5± 8.3) and PLR (179.8 ± 246.1 vs. 135 ± 64.7) than the control group. Only the mean values of all calculated lipoprotein ratios were lower in MINOCA patients. Statistical significance was achieved only in the RC evaluation. Conclusions: Higher levels of RC and MHR were found in patients with MINOCA. We also observed higher levels of PLR than in the control group. Only various lipoprotein ratios were lower, but this could reflect the extreme heterogeneity underlying the pathogenic mechanisms of MINOCA. In patients who receive a diagnosis of MINOCA with a baseline alteration of the lipid profile and higher levels of cholesterol at admission as well, the evaluation of these parameters could play an important role, providing more detailed information about their cardiometabolic risk.

## 1. Introduction

Myocardial infarction with non-obstructive coronary arteries (MINOCA) is a pathological condition due to different causes, characterized by clinical evidence of myocardial infarction (according to the Fourth Universal Definition of Myocardial Infarction) with normal or near-normal (stenosis < 50%) coronary arteries on angiography [1,2,3]. Prevalence documented in the literature is extremely variable, probably because of the lack of a unique protocol for diagnosis. In fact, around 1–15% of patients who are diagnosed with acute myocardial infarction (AMI) and who undergo coronary angiography present without any significant stenosis in their coronary arteries and receive an initial diagnosis of MINOCA [4]. From an epidemiological point of view, patients with MINOCA are generally younger (55 years vs. 61 years) and more frequently women (43% vs. 24%) than those with “classical” atherothrombotic AMI (also referred to as “type 1 myocardial infarction”). In addition, although there is a similarly high prevalence of risk factors, MINOCA seems to be less associated with hyperlipidemic disorders. On admission, MINOCA can manifest with different electrocardiographic patterns. About two-thirds of patients present with non-ST segment elevation acute coronary syndrome (NSTE-ACS), while one-third present with ST segment elevation acute coronary syndrome (STE-ACS) [5]. In contrast to the past, where MINOCA was thought to have a better short- and long-term prognosis, it is now clear that this condition is associated with unfavorable outcomes, hence the importance of a correct diagnosis and careful risk stratification of patients who belong to this clinical group [6].

As stressed by the 2017 ESC Working Group Position Paper, MINOCA should be a “working diagnosis”, that is, a starting point to clarify the etiological mechanism and achieve a definite diagnosis, in order to provide the best therapeutic option. It is even more important since this condition affects patient care and prognosis [6]. Although coronary angiography is one of the most important diagnostic tools, in most cases in the specific setting of MINOCA, it does not allow one to make a certain diagnosis. The recourse to several non-invasive (echocardiography, cardiac magnetic resonance, in particular), as well as invasive diagnostic methods, like optical coherence tomography (OCT) or intra-vascular ultrasound (IVUS) is often necessary to move forward on the diagnostic path and to make a differential diagnosis [7].

As stated in the 2023 ESC Guidelines for the management of acute coronary syndromes, MINOCA should be considered as an “umbrella” term, because it encompasses a heterogeneous group of causes. In fact, different etiologies and pathogenic mechanisms have been attributed to MINOCA. The heterogeneity of this clinical condition determines significant differences in symptom manifestations, therapeutic management and prognosis. We can schematically identify three main etio-pathological groups: (1) coronary causes (such as coronary embolism, coronary microvascular dysfunction, coronary spasm, coronary thrombosis, myocardial bridging, plaque rupture/erosion, spontaneous coronary artery dissection) (2) non-coronary cardiac causes (cardiac trauma, cardiomyopathy, cardiotoxins, myocarditis, strenuous exercise, Takotsubo cardiomyopathy, transplant rejection; (3) non-cardiac causes (acute respiratory distress syndrome, allergic/hypersensitivity reactions, end-stage renal failure, inflammation, pulmonary embolism, sepsis, stroke) [8]. Whether Takotsubo syndrome and myocarditis are causes of MINOCA, as listed here, is still a matter of debate. Some authors in fact do not include these conditions among the causes of MINOCA, and others suggest a possible microvascular involvement that could explain the development of an acute coronary syndrome (ACS) [8,9,10]. This argument, of course, deserves closer examination, but it goes far beyond the scope of this document.

Several trials demonstrated that in almost all dyslipidemic patients who undergo intensive lipid-lowering treatment (also, those who reach the “target values”), a significant residual cardiovascular risk persists [11]. This seems to be due to new independent cardiovascular risk factors, like remnant cholesterol (RC) and the monocyte/high-density lipoprotein cholesterol ratio (MHR). Their role as predictors of acute coronary events, severe coronary disease and major adverse cardiovascular events (MACEs) has been now widely highlighted [12,13]. In detail, RC corresponds to entire circulating cholesterol, without HDL-C and LDL-C [14,15]. The MHR, in addition to being a predictor of cardiovascular events, is also a systemic inflammation marker [16,17]. Along with inflammation biomarkers, we also evaluated the platelet/lymphocyte ratio (PLR), that, in addition to giving information about platelet aggregation and inflammation pathways, has been recently associated with the presence, severity and prognosis of coronary artery disease (CAD) [18]. These recently identified markers have never been evaluated in patients with MINOCA. Finally, for the same reasons, we also compared possible differences between the two groups in lipoprotein ratio values (TC/HDL-C, LDL-C/HDL-C, TG/HDL-C, and non-HDL-C/HDL-C ratios).

In our study, we wondered if the aforementioned biomarkers (RC, MHR, PLR and lipoprotein ratios) could play a decisive role in determining cardiovascular risk in the group of patients diagnosed with MINOCA, compared to a control group of patients without previous cardiovascular events.

## 2. Materials and Methods

This was a retrospective observational study whose objective was to identify predictive factors that can be associated with the risk of developing MINOCA. We focused our attention not only on classical cardiovascular risk factors, but also on RC, MHR, PLR and lipoprotein ratios.

The study population was made up of two groups. The first consisted of 114 patients who were hospitalized in the Intensive Coronary Care Unit (ICCU) and Hospital Wards of our Hospital Center from 2015 to 2019 and who were subjected to coronary angiography and received a diagnosis of MINOCA (hereinafter referred to as “MINOCA group”). The second comprised 110 patients without previous cardiovascular events but with a variable set of cardiovascular risk factors (hereafter called “control group”). In detail, patients of the control group were evaluated at cardiology clinics of our Hospital Center with comprehensive clinical, laboratory and instrumental examinations.

We developed a database containing personal data, gender, cardiovascular risk factors and comorbidities, as well as the year of hospital admission, laboratory data, ejection fraction and the evidence/absence of left ventricular hypertrophy (LVH).

Inclusion criteria were male or female sex, age above 18, clinical diagnosis of AMI according to the Fourth Definition of Myocardial Infarction [2], and absence of hemodynamically significant coronary stenoses (<50% of lumen) on coronary angiography performed during hospital stay. Conversely, patients with previous cardiovascular events, stent implantation, hemodynamically significant stenoses on coronary angiography (>50% of lumen), ongoing treatment with statins or other lipid-lowering drugs (such as exetimibe, PCSK9-inhibitors), active cancer, or active or previous lymphoproliferative disorders were excluded from the study.

Patients were considered affected by MINOCA according to the AMI criteria of the Fourth Universal Definition of Myocardial Infarction [2], in the absence of stenotic lesions > 50% of vasal lumen and in the lack of alternative diagnoses (like myocarditis or pulmonary embolism). Patients were considered smokers in cases of an active smoking habit. Arterial hypertension was defined as systolic blood pressure ≥ 140 mmHg (≥135 mmHg in case of diabetes), diastolic blood pressure ≥ 90 mmHg (≥85 mmHg if diabetes) and/or treatment with antihypertensive drugs. Metabolic syndrome was diagnosed if 3 of 5 following criteria were met according to the WHO: (1) visceral obesity (waist circumference ≥ 102 cm in men and ≥ 88 cm in women); (2) hypertriglyceridemia (>150 mg/dL); (3) HDL-C < 40 mg/dL in men and < 50 mg/dL in women; (5) blood pressure > 130/85 mmHg; (5) hyperglycemia (impaired fasting glucose > 100 mg/dL, specific pharmacological treatment or diagnosis of diabetes mellitus).

HDL-C levels < 40 mg/dL in men and <50 mg/dL in women, LDL-C > 129 mg/dL, VLDL-C > 30 mg/dL, triglycerides > 150 mg/dL and total cholesterol > 200 mg/dL were considered as altered. LDL-C levels were calculated using the Friedewald formula [LDL-C = TC − (TG/5) − HDL-C], taking into account that all patients enrolled had TG levels < 400 mg/dL. Remnant cholesterol was calculated according to the following formula: TC − HDL − LDL (including VLDL and chylomicrons). Since laboratory data were obtained from blood samples at the moment of hospitalization, we calculated non-fasting remnant cholesterol. The difference between levels of fasting and post-prandial circulating RC is irrelevant, so it is possible to perform an accurate estimation of RC levels.

The monocyte/HDL-C ratio was calculated by dividing the monocyte count of peripheral blood by HDL-C levels, while the platelet/lymphocyte ratio was obtained by dividing platelet count by lymphocyte count.

We also calculated different lipoprotein ratios, like total cholesterol/high-density lipoprotein cholesterol (TC/HDL-C), low-density lipoprotein cholesterol/high-density lipoprotein cholesterol (LDL-C/HDL-C), triglycerides/high-density lipoprotein cholesterol (TG/HDL-C), and non-high-density lipoprotein cholesterol/high-density lipoprotein cholesterol (non-HDL-C/HDL-C) ratios. Non-HDL cholesterol is present in all of the atherogenic lipoproteins (LDL-C, Lp(a), VLDL-C, IDL-C, chylomicrons and their remnants) and was calculated by subtracting the HDL-C value from total cholesterol. Finally, VLDL-C was estimated by dividing triglycerides by 5.

Data analysis was performed using R software (version 4.4.0). We used the chi-square test and student *t*-test, respectively, for qualitative and quantitative variables, in order to define the statistical significance of differences between variables taken into account. A value of *p* < 0.05 was considered indicative of statistical significance.

## 3. Results

The MINOCA group was made up of 38 men (33.3%) and 76 women (66.6%); the mean age was 57.8 ± 14.9 years. The control group consisted of 75 women (68.2%) and 35 men (31.8%), with a mean age of 57.2 ± 8.2 years. Patients with MINOCA and those in the control group had the same distribution of cardiovascular risk factors (Table 1).

We also evaluated and compared the distribution of lipid profiles between the two populations. We observed that MINOCA patients had higher mean levels of total cholesterol (165.1 ± 42.9 vs. 127.3 ± 27.7), HDL-C (50.8 ± 14.6 vs. 43 ± 6.7) and non-HDL-C (113.4 ± 43.2 vs. 111 ± 36) compared to the control group, but lower values, on average, of triglycerides (107.7 ± 52.5 vs. 112.3 ± 43.5), VLDL-C (21.3 ± 10.6 vs. 25 ± 8.5) and LDL-C (92.8 ± 38.6 vs. 104 ± 29.1). However, there were no statistically significant differences in any of these endpoints between the two groups.

**Figure 1:**
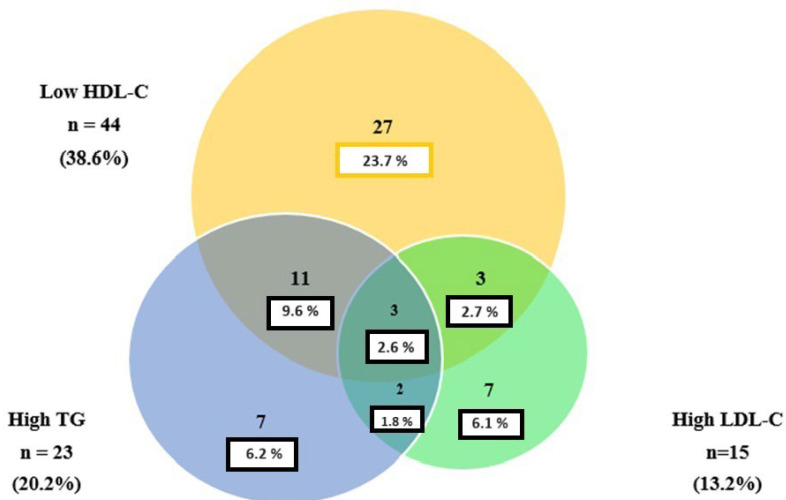
Venn diagram showing the overlaps among the prevalences of elevated values of LDL-C and triglycerides, as well as of low values of HDL-C in patients diagnosed with MINOCA.

Figure 1 presents the lipid profile of patients diagnosed with MINOCA in a Venn diagram, which shows the overlaps among the prevalences of elevated values of LDL-C as well as low values of HDL-C.

We also analyzed the ratios of lipoproteins (Table 2). It can be noted that the mean values of all calculated lipoprotein ratios (CT/HDL-C, LDL/HDL-C, TG/HDL-C and non-HDL/HDL-C) were lower in the MINOCA group than in the control group. However, these differences were not statistically significant.

Then, we focused on evaluation of remnant cholesterol (RC) and the monocyte/HDL-C and platelet/lymphocyte ratios. As shown in Table 3, patients diagnosed with MINOCA had higher values, on average, for remnant cholesterol (21.3 ± 10.6 vs. 13.2 ± 7.7 mg/dL) and the platelet/lymphocyte ratio (179.8 ± 246.1 vs. 135 ± 64.7) than the control group (Table 3).

## 4. Discussion

In this study, we focused our attention on new cardiovascular risk markers: remnant cholesterol (RC), monocyte/HDL-C ratio (MHR) and platelet/lymphocyte ratio (PLR), never evaluated before in this class of patients.

From the data reported in the literature, MINOCA patients have a high prevalence of all traditional cardiovascular risk factors, as much as those diagnosed with type 1 myocardial infarction, although dyslipidemia seems to be less common [5]. The population of our study confirmed this trend: the MINOCA group showed a high prevalence of the main cardiovascular risk factors. Moreover, compared to the control group, we observed a higher prevalence of diabetes mellitus (22% vs. 16.5%), obesity (41% vs. 13%) and metabolic syndrome (15.7% vs. 13%). This finding is not so surprising, because the relationship between these risk factors, inflammation and endothelial dysfunction is now known. In an interdependent way, diabetes mellitus and obesity cause a chronic pro-inflammatory state with increased production of circulating pro-inflammatory cytokines (interleukin-6, tumor necrosis factor-alfa, etc.) and reactive oxygen species (ROS) [19]. This leads to endothelial dysfunction and vascular remodeling, which also involves the coronary microcirculatory system [20]. Not without reason, a substantial proportion of MINOCA cases are due to coronary microvascular dysfunction, expressed as structural abnormal remodeling and/or functional vascular hyperreactivity (almost 25% of cases) [21].

As regards the distribution of lipid profiles, it was found that MINOCA patients, compared to control group, had higher mean levels of total cholesterol (TC), HDL-C and non-HDL cholesterol, but showed lower values for triglycerides, LDL-C and VLDL-C. In particular, the most significant difference between the two groups was observed in TC (165.1 ± 42.9 mg/dL in the MINOCA group vs. 127.3 ± 27.7 mg/dL in the control group). Although HDL-C mean levels in MINOCA group were above the normal cut-off (50.8 ± 14.6 mg/dL), we observed in a large number of patients low levels of HDL-C (23.7%) without other lipoprotein alteration or coexistence of high levels of triglycerides and low HDL-C (9.6%). Abnormal isolated triglycerides and LDL-C levels (6.1%) or coexistence of high LDL-C and low HDL-C (2.6%) were less frequent findings. Low values for HDL-C are an independent cardiovascular risk factor, because of the loss of their antioxidant properties. VLDL-C and triglycerides are also considered cardiovascular risk factors, since they promote atherogenesis on vascular walls. Starting from the values of lipoproteins, we decided to calculate different lipoprotein ratios. The reason for this was that several studies demonstrated that these ratios are more powerful cardiovascular risk markers than lipoproteins alone. The TC/HDL-C ratio, also known as the atherogenic index, and the LDL-C/HDL-C ratio have a similar efficacy, because about two-thirds of circulating cholesterol are contained in LDL. Values for TC/HDL-C ≥ 5.5 and LDL-C/HDL-C > 5 are associated with a higher risk of coronary events [18]. However, in our study, the MINOCA group showed lower mean values for these two ratios than those of the control group (average of values for TC/HDL-C 3.49 ± 1.3, less than the cut-off suggestive of augmented cardiovascular risk). As regards TG/HDL-C, it has been shown that this ratio is strongly correlated with the extension and severity of coronary damage. In detail, a TG/HDL-C ratio > 4 is a strong and independent predictor of coronary events [19]. The patients in our study had values for this ratio < 4. This could be due to a minor extension of coronary damage compared to MI-CAD. Non-HDL-C is the amount of cholesterol transported by LDL, Lp(a), VLDL, IDL, chylomicrons and their remnants. The HDL-C/non-HDL-C ratio seems to have predictive power for cardiovascular diseases similar to that of the TC/HDL-C and LDL-C/HDL-C ratios [20]. We observed lower mean values for the non-HDL-C/HDL-C ratio in the MINOCA group compared to the control group. Therefore, patients with MINOCA in our study had lower mean levels of overall lipoprotein ratios evaluated than those in the control group. These results could be related to the great heterogeneity of pathophysiological mechanisms underlying MINOCA. We have already pointed out the importance of considering MINOCA as a “working diagnosis”. Probably in specific subgroups of patients, the lipid profile alterations play a more important role in the pathogenesis of this condition, while in others, the lipid profile is normal, and this could suggest a different cause. In our opinion, the most important mechanism of MINOCA is the rupture/erosion of atheromatous plaque (about 40% of cases), since it has been demonstrated that the rupture of plaques with a modest content of lipids is possible, especially in a context of different thrombogenic factors [21,22]. We also evaluated remnant cholesterol and the monocyte/HDL ratio, new markers of cardiovascular risk. In some studies, it has been shown that a progressive increase in non-fasting RC levels is significantly related to an increased risk of coronary events and overall mortality. Patients with RC values > 43 mg/dl had a 2–3 fold increased risk of coronary artery disease com-pared to subjects with RC values < 15 mg/dL [15,23]. In this study, the MINOCA group had higher mean non-fasting RC levels compared to the control group (21.3 ± 10.6 vs. 13.2 ± 7.7).

This result supports what we said earlier, that is, patients who had a previous cardiovascular event (MINOCA in this specific case) have higher non-fasting RC circulating levels that are associated with a high risk of cardiovascular events. These results, moreover, had statistical significance (*p* = 0.0001). As regards the analysis of the monocyte/HDL ratio (MHR), our study showed higher mean levels in MINOCA patients compared to the control group (23 ± 0.009 vs. 18.5 ± 8.3), although this difference did not reach statistical significance (*p* = 0.05). MHR is a new prognostic marker of cardiovascular risk, directly related to inflammation and oxidative stress. This marker demonstrated its utility as an independent predictor of in-hospital mortality and MACEs in patients with STEMI [24]. Several studies confirm the usefulness of this marker for the prediction of cardiovascular risk. A cut-off (12.1) has been identified, above which we can carefully predict the presence of coronary stenoses [25]. It should be noted that all of the parameters analyzed here could vary in different ethnic groups. Our group showed that there are differences in RC and MHR values between Caucasian and South Asian patients [26]. The last ratio evaluated was the PLT/lymphocyte ratio. Although the differences between the two groups were not statistically significant (*p* = 0.05), MINOCA patients had higher mean values compared to those in the control group (179.8 ± 246.1 vs. 135 ± 64.7). This marker, in fact, provides information about platelet aggregation pathways and inflammation and could be more valid in the prediction of cardiovascular risk and coronary damage than the platelet or lymphocytic counts considered separately.

## 5. Conclusions

In this study, we observed higher remnant cholesterol levels and monocyte/HDL-C ratios in a group of patients diagnosed with MINOCA compared to a control group of patients without previous cardiovascular events. Moreover, we also highlighted higher platelet/lymphocyte ratios compared to the control group. Only the lipoprotein ratios in the MINOCA group were lower. This could reflect the extreme heterogeneity underlying the pathogenetic mechanisms of MINOCA, suggesting a minor role of dyslipidemia in this group of patients compared to those diagnosed with MI-CAD.

In patients who receive a diagnosis of MINOCA with a baseline alteration of the lipid profile and higher levels of cholesterol at admission as well, the evaluation of these parameters could play an important role, providing more detailed information about their cardiometabolic risk. Because of the countless causes related to MINOCA, it should be noted that the therapeutic approach to this condition is very heterogeneous. The use of these specific markers (very simple to calculate and almost entirely inexpensive) could help clinicians to better identify those patients that may benefit the most from a stronger and more careful lipid-lowering therapy. In particular, patients with higher remnant cholesterol levels and monocyte/HDL-C ratios could benefit from intensive treatment with cholesterol-lowering strategies. However, further studies with larger samples are needed to validate the assessment of these parameters in clinical practice and in patients with MINOCA in particular.

## 6. Study Limitations and Future Perspectives

Our study has some limitations. First, this was a retrospective observational study with the inherent limitations of such a study. Second, the number of enrolled patients was too small. Many patients could not be included, as they did not meet the inclusion criteria, mainly due to the lack of important data to conduct our study. Third, the features of the control group could represent a limitation of our study, because it included patients without previous cardiovascular events compared to patients diagnosed with AMI. In the future, it would be interesting to compare MINOCA patients with a control group of patients diagnosed with “classical” atherothrombotic AMI.

## Figures and Tables

**Table 1:** Comparison of demographic and clinical characteristics between MINOCA patients and the control group.

	MINOCA Group (n = 114)	Control Group (n = 110)	*p*-Value
Age (mean age ± SD)	57.8 ± 10.9	57.2 ± 8.2	0.05
Hypertension n (%)	73 (64)	44 (45)	0.82
Diabetes n (%)	25 (22)	18 (16.5)	0.03
Obesity n (%)	27 (41)	16 (13)	0.06
Metabolic syndrome n (%)	18 (15.7)	15 (13)	0.03
Hypercholesterolemia n (%)	38 (29)	24 (30)	<0.06
Hypertriglyceridemia n (%)	18 (15.7)	9 (8.5)	0.06
Smoking history n (%)	15 (13)	45 (43)	0.0001
Family history n (%)	17 (17.4)	19 (18)	0.7

**Table 2:** Comparison of values for lipoprotein ratios between the MINOCA group and control group.

	MINOCA Group (n = 114)		Control Group (n = 110)		*p*-Value
	M	SD	M	SD	
TC/HDL-C	3.49 (2–10.13)	1.3	4.6 (2.6–8.1)	1.2	0.38
LDL-C/HDL-C	2 (0.25–8.07)	1.1	3.5 (1.5–6.6)	1.8	0.71
TG/HDL-C	2.46 (0.45–9.13)	1.7	3.7 (1.5–9.6)	1.5	0.15
Non-HDL-C/HDL-C	2.49 (0.42–6)	1.3	3.9 (1.2–7.3)	1.4	0.38

**Table 3:** Comparison of values for remnant cholesterol (RC) and monocyte/HDL-C and platelet/lymphocyte ratios between the MINOCA group and control group. M = average values (minimum and maximum values of the series in brackets); SD = standard deviation; RC = remnant cholesterol; PLT = platelets.

	MINOCA Group (n = 114)		Control Group (n = 110)		*p*-Value
	M	SD	M	SD	
RC (mg/dL)	21.3 (6.4–60.6)	10.6	13.2 (11–57)	7.7	0.0001
Monocytes (×10^3^/mm^3^)	1.1 (0.5–2.11)	0.2	0.5 (0.03–1.2)	0.1	0.47
Monocyte/HDL-C	23 (18.3–26.5)	0.009	18.5 (0.7–51)	8.3	0.05
PLT (×10^3^/mm^3^)	257.9 (116–565)	78.4	227 (129–396)	58	0.18
PLT/lymphocyte	179.8 (38.4–2560)	246.1	135 (57–293)	64	0.05

## Data Availability

Data are contained within the article.
